# The Potential of Alamandine in Airway Hyperresponsiveness in an Ovalbumin-Induced Asthma Model

**DOI:** 10.3390/biomedicines14081832

**Published:** 2026-08-14

**Authors:** Vitória Nedel Rech, Andresa Thomé Silveira, Giuliano Rizzotto Guimarães, Katya Rigatto

**Affiliations:** 1 Laboratório de Fisiologia Translacional, Universidade Federal de Ciências da Saúde de Porto Alegre (UFCSPA), Porto Alegre 90050-170, Brazil; vitoria.rech@ufcspa.edu.br (V.N.R.); andresasilveira@ufcspa.edu.br (A.T.S.); 2 Programa de Pós-Graduação em Ciências da Saúde, Universidade Federal de Ciências da Saúde de Porto Alegre (UFCSPA), Porto Alegre 90050-170, Brazil; 3 Laboratório de Patologia, Universidade Federal de Ciências da Saúde de Porto Alegre (UFCSPA), Porto Alegre 90050-170, Brazil; giulianog@ufcspa.edu.br

**Keywords:** asthma, alamandine, renin–angiotensin system, MrgD receptor, airway hyperresponsiveness, ovalbumin model

## Abstract

**Background**: Asthma is a chronic inflammatory airway disease characterized by variable airflow obstruction and tissue remodeling. Alamandine (ALA), a renin–angiotensin system (RAS) peptide with anti-inflammatory properties, remains poorly explored in allergic respiratory disease. **Objectives:** This study evaluated prophylactic (33 days) and therapeutic (21 days) subcutaneous ALA (50 µg/kg/day) administration in an ovalbumin (OVA)-induced rat asthma model. **Methods**: Twenty male Wistar rats were assigned to four groups: control (CO), OVA with prophylactic ALA (ALA-P; days 1–33), OVA with therapeutic ALA (ALA-T; days 12–33), and OVA-only control (OVA). Rats were sensitized (days 1–3) and challenged with aerosolized OVA (days 6–33). On day 34, ventilatory mechanics were assessed via FlexiVent, including methacholine challenge (12.5 mg/mL). Pulmonary inflammation was evaluated by eosinophil quantification; MrgD/Mas and AT1 receptor expression by immunohistochemistry and Western blot, respectively. **Results**: OVA animals exhibited elevated baseline airway resistance and bronchoconstriction, and eosinophil infiltration. ALA-T significantly attenuated the respiratory symptoms, restoring airway resistance and eosinophil infiltration to CO-group levels, whereas ALA-P exhibited only a partial reduction. MrgD and Mas expression were elevated in OVA, reflecting compensatory RAS activation, but normalized in both ALA-treated groups. No significant differences in AT1 expression were observed. **Conclusions**: ALA likely exerts bronchoprotective and anti-allergic effects in this model via MrgD/Mas modulation. The ALA–MrgD/Mas axis represents a potential therapeutic target for allergic airway diseases, particularly when conventional treatments are insufficient.

## 1. Introduction

Asthma is a complex airway disease involving multiple cell types. It is characterized by variable airflow obstruction, chronic inflammation, airway remodeling, and hyperresponsiveness (AHR). Its nonspecific symptoms often overlap with other causes of cough or dyspnea [1,2,3].

According to the World Health Organization, the global burden of asthma reached 262 million cases in 2019. The disease caused approximately 455,000 deaths that year. As a significant noncommunicable disease, asthma remains central to global efforts to achieve a one-third reduction in premature mortality by 2030 [4,5,6].

Asthma leads to expiratory flow limitation, causing air trapping and dynamic pulmonary hyperinflation. Airway resistance is highly sensitive to reductions in airway caliber. Even modest bronchoconstriction can substantially increase the pressure required to sustain airflow, leading to ventilatory constraint [7,8]. These functional changes are driven by airway remodeling, including thickening of the reticular basement membrane and structural alterations in both large and small airways.

In the T2-high (eosinophilic/allergic) asthma endotype, these manifestations are closely associated with a type 2 immune response. Inflammatory infiltrates, particularly CD4+ T-helper lymphocytes with type 2 (Th2)-skewed responses, promote eosinophilic inflammation. This inflammation correlates positively with AHR and disease severity [9,10,11]. Notably, eosinophils express Mas receptors [12,13,14]. This suggests that the ALA–MrgD–Mas axis may play a role in eosinophilic recruitment and activation. Therefore, eosinophil infiltration serves as a relevant functional readout of counter-regulatory RAS activity in allergic airway inflammation.

Current management relies primarily on bronchodilators and inhaled corticosteroids (ICS) to achieve symptom control and reduce exacerbation risk [15]. However, conventional ICS therapy is not effective for all patients. Long-term use is associated with significant adverse effects, including reduced bone mineral density, suppression of the hypothalamic–pituitary–adrenal (HPA) axis, growth impairment, oral candidiasis, and dysphonia [16,17,18,19].

In recent years, major advances in asthma treatment have occurred with the approval of numerous biologic therapies. These have resulted in substantial reductions in exacerbations, improved lung function, and enhanced quality of life for patients with severe asthma. Despite these significant advances with biologics, important unmet needs remain in severe asthma treatment. These include incomplete responses in patients with high type 2 inflammation, limited options for low type 2 asthma, and a lack of predictive biomarkers. Given these impactful limitations, new therapeutic strategies are still urgently required [20,21].

In this context, the renin–angiotensin system (RAS), classically known for its pulmonary activation, has emerged as a potential modulator of respiratory pathophysiology. The Mas receptor is expressed in airway epithelial and smooth muscle cells [22,23,24], as well as in eosinophils and alveolar macrophages [12,13,14]. Furthermore, allergic inflammation significantly reduces pulmonary ACE2 expression [25,26]. This suggests that the counter-regulatory RAS axes, specifically ACE2–Ang-(1-7)–Mas and ACE2–alamandine (ALA)–MrgD, may play a role in asthma pathophysiology.

Recent results in the human lung showed that Mas and MrgD receptors exhibit differential distribution. Mas predominates in bronchial regions, while MrgD is more prominently expressed in the parenchyma. Both receptors show altered expression in interstitial lung diseases, suggesting distinct yet complementary roles in pulmonary structure and function [24].

Emerging evidence indicates that the biological effects of ALA depend on a complex interplay between Mas and MrgD receptors. This supports the concept that these receptors act as an integrated network rather than independently [27]. The functional cooperation, potentially involving heterodimerization between Mas and MrgD [28,29], provides a mechanistic basis for interpreting receptor expression patterns observed in immunohistochemical analyses.

Moreover, ALA has demonstrated potent anti-inflammatory [30,31,32,33], antifibrotic [34,35,36,37], anti-apoptotic [33,35,38], and antioxidant [33,34,38] effects across various tissues, particularly within the cardiovascular system [30,31,32,33,34,35,36,37,38]. These pleiotropic actions involve multiple mechanisms, including modulation of MAPK signaling pathways and nitric oxide (NO) production [39]. Despite the therapeutic potential of ALA, its role in allergic respiratory diseases remains poorly explored, particularly regarding the optimal timing of intervention.

By comparing prophylactic and therapeutic administration, this study aims to elucidate whether the participation of the ALA–MrgD–Mas axis differs depending on the stage of disease. Evaluating receptor expression across different treatment timelines is essential to determine whether ALA’s anti-inflammatory and bronchoprotective properties are more effectively harnessed for prevention or for reversing established disease.

Given that receptor expression, including that of AT1 and Mas, is highly sensitive to disease temporal progression and tissue compartment [24,39], this approach provides a more nuanced interpretation of RAS signaling. It also allows for the identification of functional shifts in the counter-regulatory axes that may occur even when classical receptor levels remain unchanged. Collectively, these findings suggest that ALA could represent a promising targeted therapeutic strategy for allergic asthma. Further studies are warranted to confirm its clinical potential.

## 2. Materials and Methods

All experimental procedures were approved by the Animal Use Ethics Committee of the Universidade Federal de Ciências da Saúde de Porto Alegre (UFCSPA), under protocol number 596/2023. Procedures were conducted in accordance with ethical standards. Twenty male Wistar rats (initial body weight: 280 ± 20 g; n = 5 per group) were obtained from the UFCSPA animal facility and randomly assigned to four groups: CO (control), OVA (asthma), ALA P (preventive ALA), and ALA T (therapeutic ALA).

Wistar rats were selected based on their established use in our laboratory for cardiovascular and respiratory physiology research. This strain reliably develops airway inflammation and hyperresponsiveness when sensitized with an aluminum hydroxide-adjuvanted OVA protocol [40], although the genetic background accounts for only part of the variability in allergic response [41].

Multiple studies have shown that Wistar and other outbred rat strains, when appropriately sensitized with OVA and aluminum hydroxide adjuvant, reliably develop the key hallmarks of allergic asthma [42,43,44,45]. Strain-related differences in response magnitude are thought to reflect disease heterogeneity rather than model invalidity. Comparative studies indicate that non-Th2-predisposed outbred strains still develop eosinophilic inflammation, elevated IgE, and adhesion molecule upregulation upon OVA sensitization [41,46]. This suggests that Wistar rats may model a phenotype closer to the genetically heterogeneous human asthmatic population rather than to highly atopic individuals alone.

### 2.1. Asthma Induction

**Figure 1:**
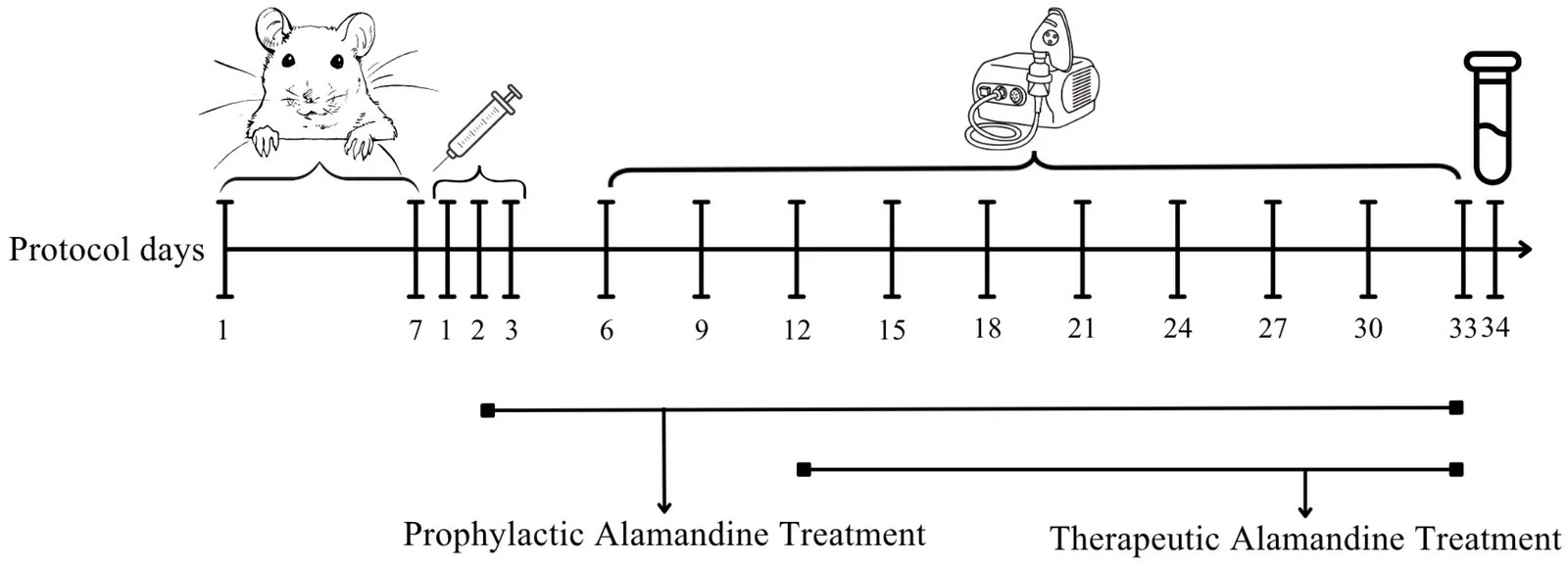
Experimental  protocol timeline for alamandine treatment in an OVA-induced asthma rat model. Icons represent the following experimental phases: rat illustration = acclimatization period; syringe = intraperitoneal sensitization injections; nebulizer = OVA inhalation challenge sessions; collection tube = sample collection and experimental endpoint. Animals underwent a 7-day acclimatization period (days 1–7). On days 1, 2, and 3 of the protocol (after acclimatization), OVA groups (OVA, ALA-P, and ALA-T) received intraperitoneal injections of OVA (1 mg) adsorbed to aluminum hydroxide (50 mg) in 0.5 mL saline for sensitization; the control (CO) group received saline only. From day 6 to day 33 (days 6, 9, 12, 15, 18, 21, 24, 27, 30, and 33), asthmatic groups were challenged with aerosolized 2% OVA in saline for 30 min in ventilated chambers, while CO animals inhaled saline under identical conditions. The prophylactic alamandine (ALA-P) group received daily subcutaneous injections of alamandine (50 µg/kg/day) from day 1 to day 33, whereas the therapeutic alamandine (ALA-T) group received the same regimen from day 12 to day 33. The CO and OVA groups received equivalent daily subcutaneous injections of saline throughout the protocol. Animals were euthanized, and samples were collected on day 34.

After a 7-day acclimatization period, a 33-day OVA-induced asthma protocol was implemented (Figure 1), adapted from Mahmoudabady et al. (2013) [40]. On days 1, 2, and 3, OVA-induced asthma groups (OVA, ALA-P, and ALA-T) received intraperitoneal injections containing 1 mg OVA (Sigma-Aldrich, St. Louis, MO, USA) and 50 mg aluminum hydroxide in 0.5 mL saline (NaCl 0.9%). CO animals received saline only (0.5 mL).

From day 6 to day 33 (on days 6, 9, 12, 15, 18, 21, 24, 27, 30, and 33), the asthmatic groups were exposed to aerosolized 2% OVA diluted in 0.9% NaCl for 30 min inside ventilated transparent chambers. CO animals inhaled saline under the same conditions. Rats in the prophylactic group (ALA-P) received daily subcutaneous injections of ALA (50 µg/kg/day) from day 1 to day 33. Rats in the therapeutic group (ALA-T) received the same dose from day 12 to day 33. ALA was dissolved in saline vehicle and administered once daily. To standardize procedures, animals in the CO and OVA groups received daily subcutaneous injections of equivalent volumes of saline. The sensitization schedule and the 3-day interval between the final sensitization and challenge onset are consistent with previously validated OVA-induced protocols in Wistar and other outbred rat strains, in which comparable or shorter intervals were sufficient to establish airway hyperresponsiveness and eosinophilic inflammation [47,48].

### 2.2. Ventilatory Mechanics (Day 34)

On day 34, 24 h after the final aerosolized OVA challenge, animals were anesthetized with ketamine (90 mg/kg) and xylazine (10 mg/kg), positioned supine, and tracheostomized to allow for the insertion of a 2 mm internal-diameter cannula connected to a FlexiVent mechanical ventilator (SCIREQ, Montreal, QC, Canada). Animals were ventilated at a tidal volume of 10 mL/kg and respiratory rate of 90 breaths/min, with positive end-expiratory pressure (PEEP) of 3 cmH_2_O. Airway resistance (Rn), static compliance (Cst), and elastance (Est) were assessed using the Prime-8 broadband perturbation and SnapShot-90 maneuver, respectively. Subsequently, animals inhaled aerosolized methacholine (acetyl-β-methylcholine chloride, 12.5 mg/mL; Sigma-Aldrich) for 3 min, after which the same parameters were reassessed to determine airway hyperresponsiveness.

### 2.3. Tissue Collection

After ventilatory assessment, animals were euthanized by anesthetic overdose. The left lung was inflated with 10% buffered formalin, removed, and immersed in formalin for 24 h. Tissue samples were then processed through a graded ethanol series, cleared in xylene, and embedded in paraffin. Sections of 4 µm thickness were cut and mounted on silanized slides for subsequent histological and immunohistochemical analyses. The right lung was collected separately, snap-frozen, and stored at −80 °C for subsequent biochemical analyses.

### 2.4. Histology with HE Staining

Following fixation in 10% buffered formalin and paraffin embedding, lung tissue was sectioned at a thickness of 5 µm and prepared for hematoxylin and eosin (H&E) staining. The sections were deparaffinized and rehydrated through a graded alcohol series to distilled water, and then stained with Harris hematoxylin for 6 to 15 min. Subsequently, slides were washed in running water for 5 min, differentiated in 1% acid alcohol using two brief immersions, and rinsed again. Nuclei were blued in a weak ammoniacal solution until intense blue coloration was achieved, followed by a final 10 min rinse in running water.

For cytoplasmic staining, the slides were immersed in 80% ethyl alcohol for 2 min and then counterstained with eosin–phloxine for an additional 2 min. Dehydration and clearing were performed through sequential 2 min baths in 90% ethyl alcohol, absolute alcohol, and xylene. Finally, the sections were mounted using a resin-based medium for subsequent microscopic evaluation.

### 2.5. Evaluation of Inflammation by Inflammatory Cells

Following H&E staining, lung tissue sections were examined to assess the inflammatory response in all experimental groups. Eosinophils, key inflammatory cells in allergic asthma, were quantified in 10 randomly selected, non-overlapping fields per slide. This analysis enabled a standardized assessment of pulmonary inflammation, allowing for quantification of eosinophilic infiltrates and comparison of inflammatory burden across treatment groups.

### 2.6. Immunohistochemistry

Immunohistochemical analysis was performed on 3 µm serial sections from formalin-fixed, paraffin-embedded (FFPE) lung tissue. Sections were mounted on labeled slides and incubated at 50 °C for 24 h to ensure tissue adherence. They were then deparaffinized in xylene and rehydrated through a graded alcohol series to distilled water.

Heat-induced antigen retrieval (HIAR) was performed using a preheated Tris-EDTA solution (pH 9.0) in a water bath at 98 °C for 40 min. Slides were then gradually cooled to room temperature and washed in PBS containing 0.05 M NaCl, 0.02 M sodium monophosphate monohydrate (NaH_2_PO_4_·H_2_O), and 0.08 M sodium diphosphate dihydrate (Na_2_HPO_4_·2H_2_O), pH 7.4. Endogenous peroxidase activity was blocked with 5% hydrogen peroxide in methanol (three 10 min cycles). Nonspecific binding was minimized by incubation with 1% bovine serum albumin (BSA) in PBS for 1 h at room temperature.

Sections were then incubated overnight at 4 °C in a humidified chamber with primary antibodies (1:200 dilution) against Mas (NBP1-60091, Novus Biologicals, Centennial, CO, USA) and MrgD (ab155099, Abcam, Cambridge, UK). Following thorough PBS washes, the sections were incubated with a polymer-based peroxidase-conjugated secondary antibody (DAKO EnVision™, REF K8000, Dako Denmark ApS, Glostrup, Denmark) for 40 min at room temperature.

The chromogenic signal was developed using 3,3′-diaminobenzidine (DAB) according to the manufacturer’s instructions. Careful monitoring to prevent excess background staining. Slides were then counterstained with hematoxylin, dehydrated through an alcohol series, cleared in xylene, and mounted with synthetic resin-based medium. For quantification, ten randomly selected fields per slide were captured and analyzed using Image Lab™ Software (version 6.0, Bio-Rad Laboratories, Hercules, CA, USA), with five animals per group (n = 5).

### 2.7. Western Blot

Pulmonary tissue was snap-frozen in liquid nitrogen and stored at −80 °C for subsequent analysis of AT1 receptor protein expression via Western blotting. To ensure equal protein loading, 75 μg of protein per sample was resolved by one-dimensional sodium dodecyl sulfate-polyacrylamide gel electrophoresis (SDS-PAGE) using a 10% resolving gel within a discontinuous system.

Following electrophoresis, the separated proteins were electrotransferred onto PVDF membranes using ice-cold transfer buffer (20 mM Tris, 150 mM glycine, 20% *v*/*v* methanol, and 0.02% *w*/*v* SDS; pH 8.2) in a chilled transfer unit (Bio-Rad, Hercules, CA, USA). Nonspecific binding sites were then blocked by incubating the membranes with 2% BSA in PBS for 1 h at room temperature.

The membranes were probed with a primary polyclonal antibody against the angiotensin II AT1 receptor (Enzo Life Sciences, Farmingdale, NY, USA). Subsequently, the membranes were incubated with a horseradish peroxidase (HRP)-conjugated goat anti-rabbit IgG secondary antibody (Sigma-Aldrich), and immunoreactive bands were visualized using enhanced chemiluminescence. Band intensities were quantified by densitometry using Image Lab™ Software (version 6.0) and a ChemiDoc™ MP Imaging System, Gel Doc™, Bio-Rad Laboratories, Hercules, CA, USA. Results were normalized to total protein load to ensure accurate relative quantification.

### 2.8. Statistical Analysis

Data normality was assessed using the Shapiro–Wilk test. All continuous variables met the assumption of normality. Differences among the four experimental groups across all parameters—including ventilatory mechanics, eosinophil, Western blot quantification of AT1 expression, and immunohistochemical quantification of MrgD and Mas expression—were analyzed using one-way ANOVA followed by Tukey’s post hoc test for multiple comparisons. Data are presented as mean ± standard deviation (SD). Statistical significance was defined as *p* ≤ 0.05.

## 3. Results

### 3.1. Ventilatory Mechanics

Airway resistance ($R_{n}$) increased significantly from pre- to post-methacholine challenge only in the ALA-P (p=0.020) and OVA (p<0.001) groups. No significant pre-to-post change was observed in the CO or ALA-T groups, indicating that effective methacholine-induced bronchoconstriction was not uniform across conditions.

No significant differences in baseline (pre-methacholine) $R_{n}$ were found between OVA and either CO (p=0.682) or ALA-T (p=0.991), indicating that airway hyperresponsiveness had not yet emerged at baseline in this model.

**Figure 2:**
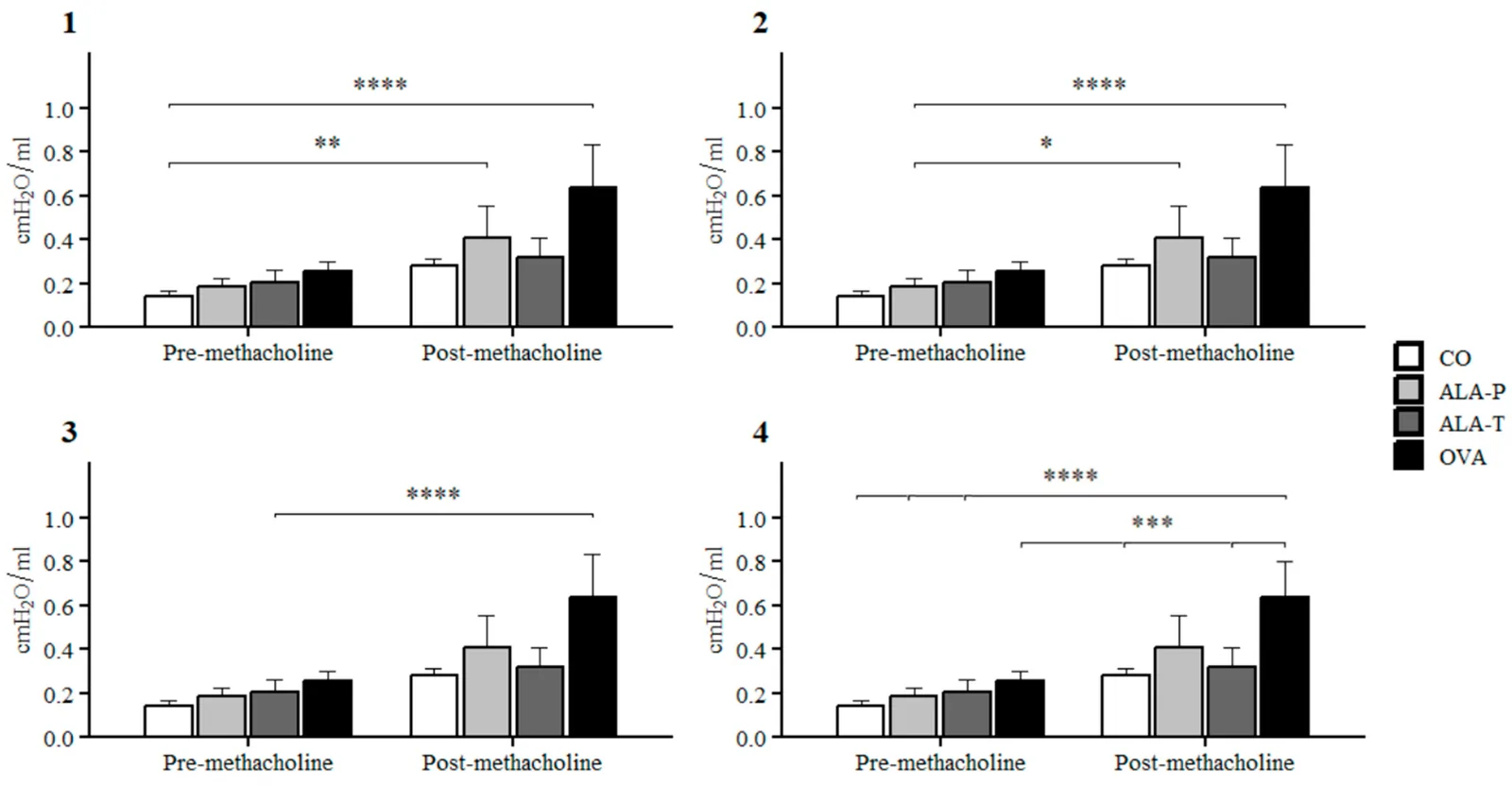
Airway resistance ($R_{n}$) between different moments. Airway resistance (${\mathrm{cmH}}_{2}O/\mathrm{mL}$) measured before (pre-methacholine) and after (post-methacholine) bronchoprovocation challenge in four experimental groups: CO (control), ALA-P (prophylactic alamandine treatment), ALA-T (therapeutic alamandine treatment), and OVA (ovalbumin-sensitized). Data are expressed as mean ± SD (n=5 per group). Statistical differences were assessed by one -way ANOVA followed by Tukey’s post hoc test. For clearer visualization of group-to-group contrasts, data are presented across four panels, each highlighting comparisons relative to a single reference group: CO (**panel 1**), ALA-P (**panel 2**), ALA-T (**panel 3**), and OVA (**panel 4**). * *p* < 0.05; ** *p* < 0.01; *** *p* < 0.001; **** *p* < 0.0001.

Following methacholine challenge, the OVA group showed significantly greater $R_{n}$ than CO (p<0.001), ALA-T (p<0.001), and ALA-P (p=0.026). OVA post-challenge $R_{n}$ was also significantly higher than the pre-challenge baseline of every other group (all p<0.0001), underscoring the magnitude of the OVA-induced response (Figure 2(4)). In contrast, the ALA-T group did not differ significantly from CO at either timepoint (baseline: p=0.973; post-challenge: p=0.999), consistent with therapeutic ALA treatment preventing an exaggerated post-challenge response (Figure 2(1),(3)). The ALA-P group’s post-challenge $R_{n}$ was significantly higher than its own baseline (p=0.020) and than the pre-challenge baseline of CO (p=0.006) and ALA-T (p=0.039), suggesting that prophylactic ALA administration did not fully prevent an increase in airway resistance after challenge, though its post-challenge value was less pronounced than OVA’s (Figure 2(2)).

No significant differences were observed in static compliance or elastance at baseline nor post-methacholine challenge across any of the groups.

### 3.2. Inflammatory Cells

**Figure 3:**
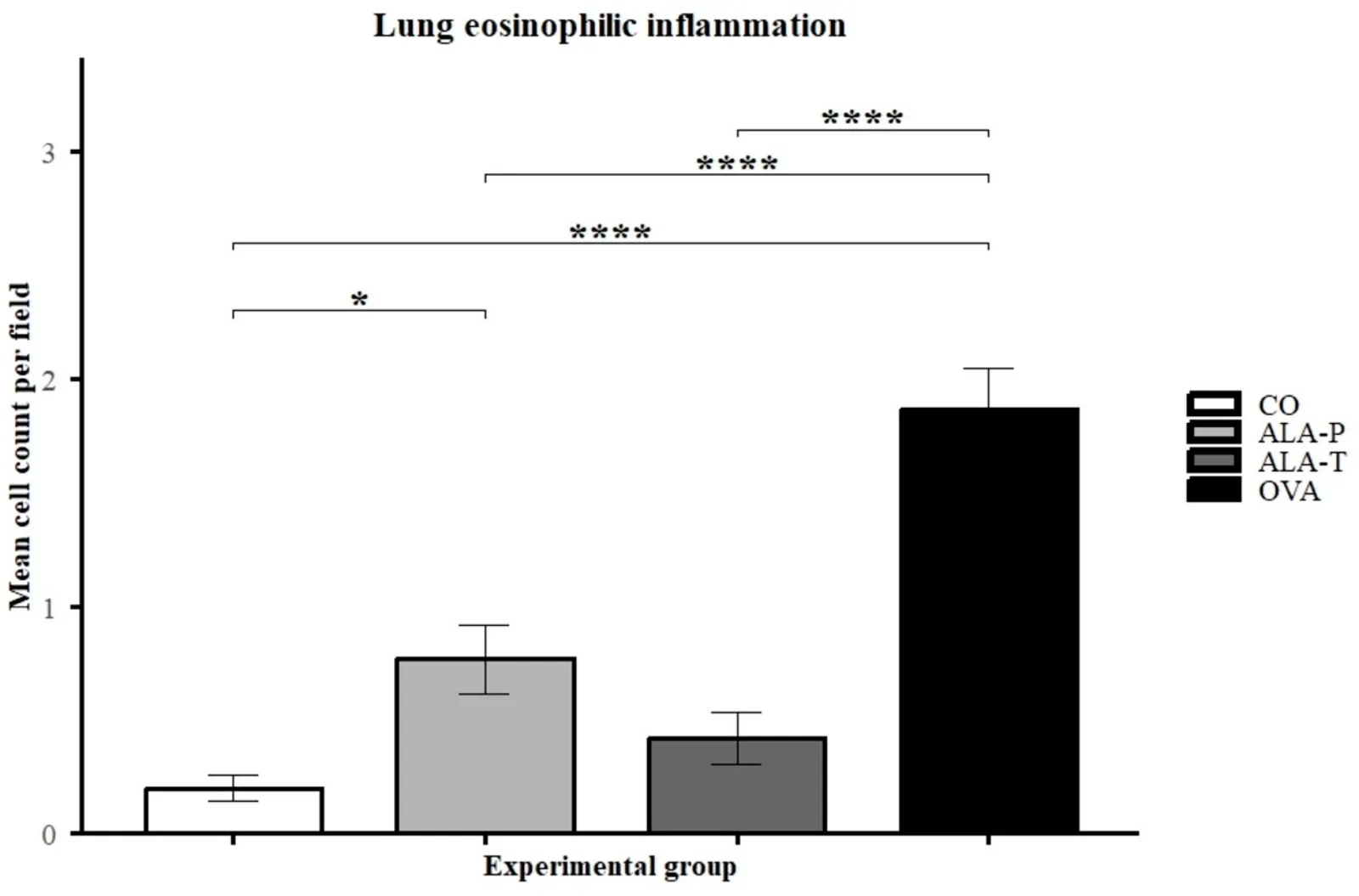
Mean number of eosinophils per field in the different experimental groups. CO: control group; ALA-P: prophylactic alamandine-treated group; ALA-T: therapeutic alamandine-treated group; OVA: ovalbumin-sensitized group. Five animals were analyzed per group. Bars represent mean ± SD. Differences between groups were assessed by one-way ANOVA followed by Tukey’s post hoc test. * *p* < 0.05; **** *p* < 0.0001.

The OVA group exhibited a significant increase in eosinophil counts compared to all other experimental groups (*p* < 0.0001; Figure 3), confirming successful induction of eosinophilic inflammation. Notably, ALA-T treatment reversed this inflammatory response: eosinophil counts in the ALA-T group were significantly lower than in the OVA group (*p* < 0.0001) and were restored to levels comparable to both the CO group (*p* = 0.724) and the ALA-P group (*p* = 0.317). These results demonstrate that therapeutic ALA administration effectively reversed asthma-induced eosinophilic inflammation. In contrast, although prophylactic ALA-P treatment also significantly reduced eosinophil infiltration compared to the OVA group (*p* < 0.0001), it did not fully restore baseline levels, as counts remained significantly elevated relative to the CO group (*p* = 0.028).

### 3.3. Immunohistochemistry

**Figure 4:**
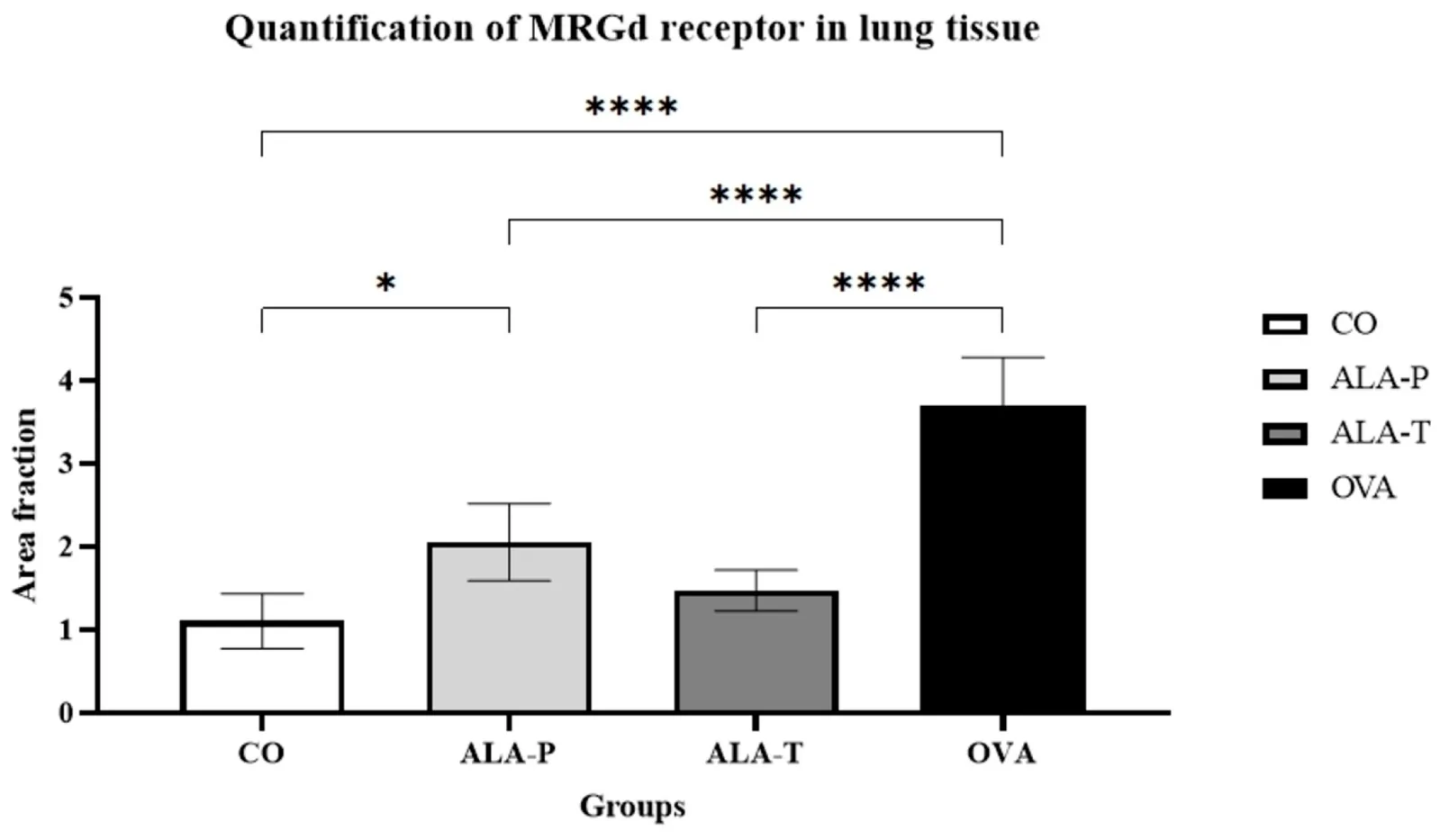
Immunohistochemical quantification of the fraction of area positive for the MrgD receptor in lung tissue across experimental groups. CO: control group; ALA-P: prophylactic alamandine-treated group; ALA-T: therapeutic alamandine-treated group; OVA: ovalbumin-sensitized group. Bars represent the mean ± SD. Differences between groups were analyzed by one-way ANOVA followed by Tukey’s post hoc test. * *p* < 0.05; **** *p* < 0.0001.

**Figure 5:**
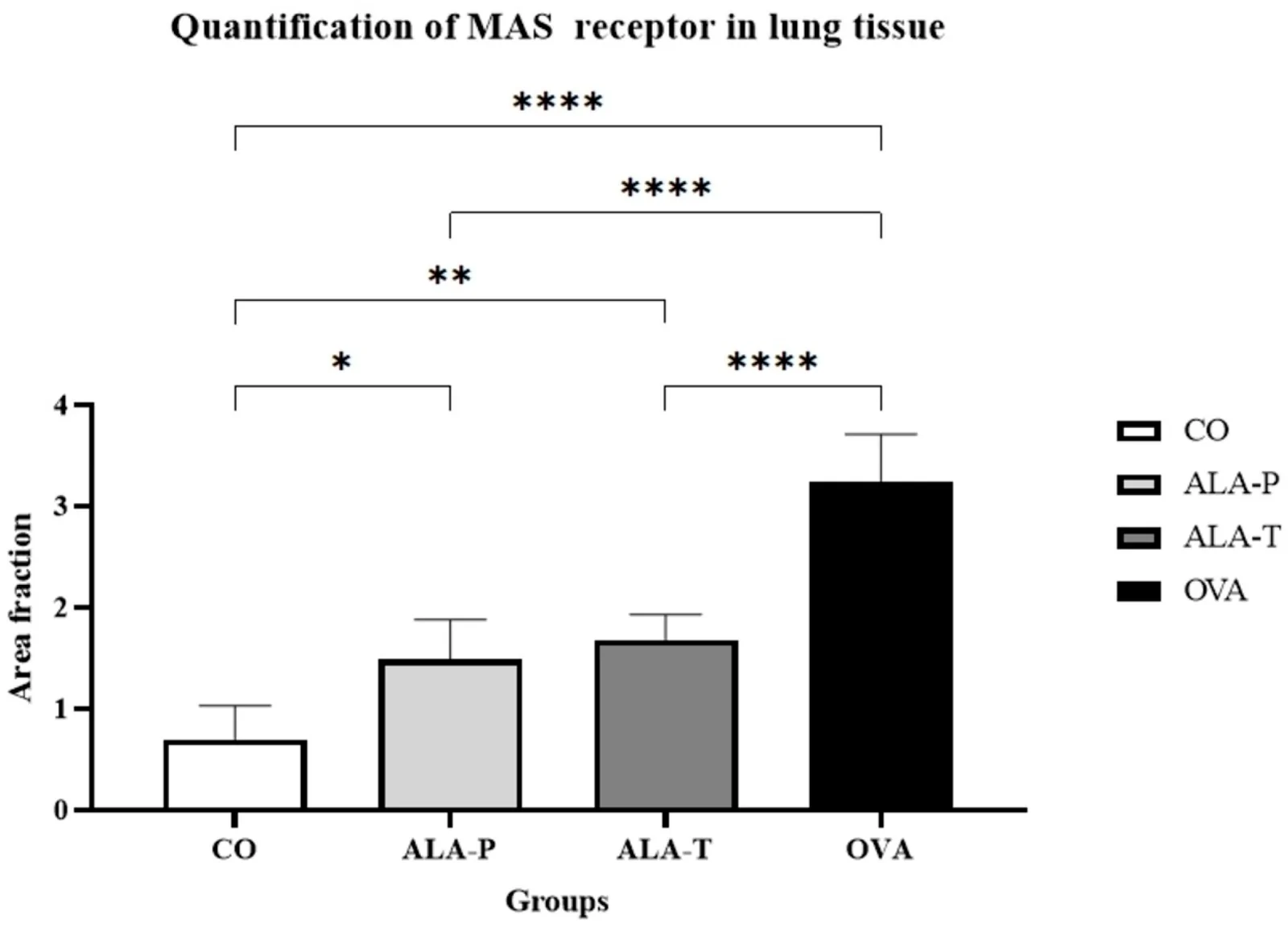
Immunohistochemical quantification of the fraction of area positive for the Mas receptor in lung tissue across experimental groups. CO: control group; ALA-P: prophylactic alamandine-treated group; ALA-T: therapeutic alamandine-treated group; OVA: ovalbumin-sensitized group. Bars represent the mean ± SD. Differences between groups were analyzed by one-way ANOVA followed by Tukey’s post hoc test. * *p* < 0.05; ** *p* < 0.01; **** *p* < 0.0001.

**Figure 6:**
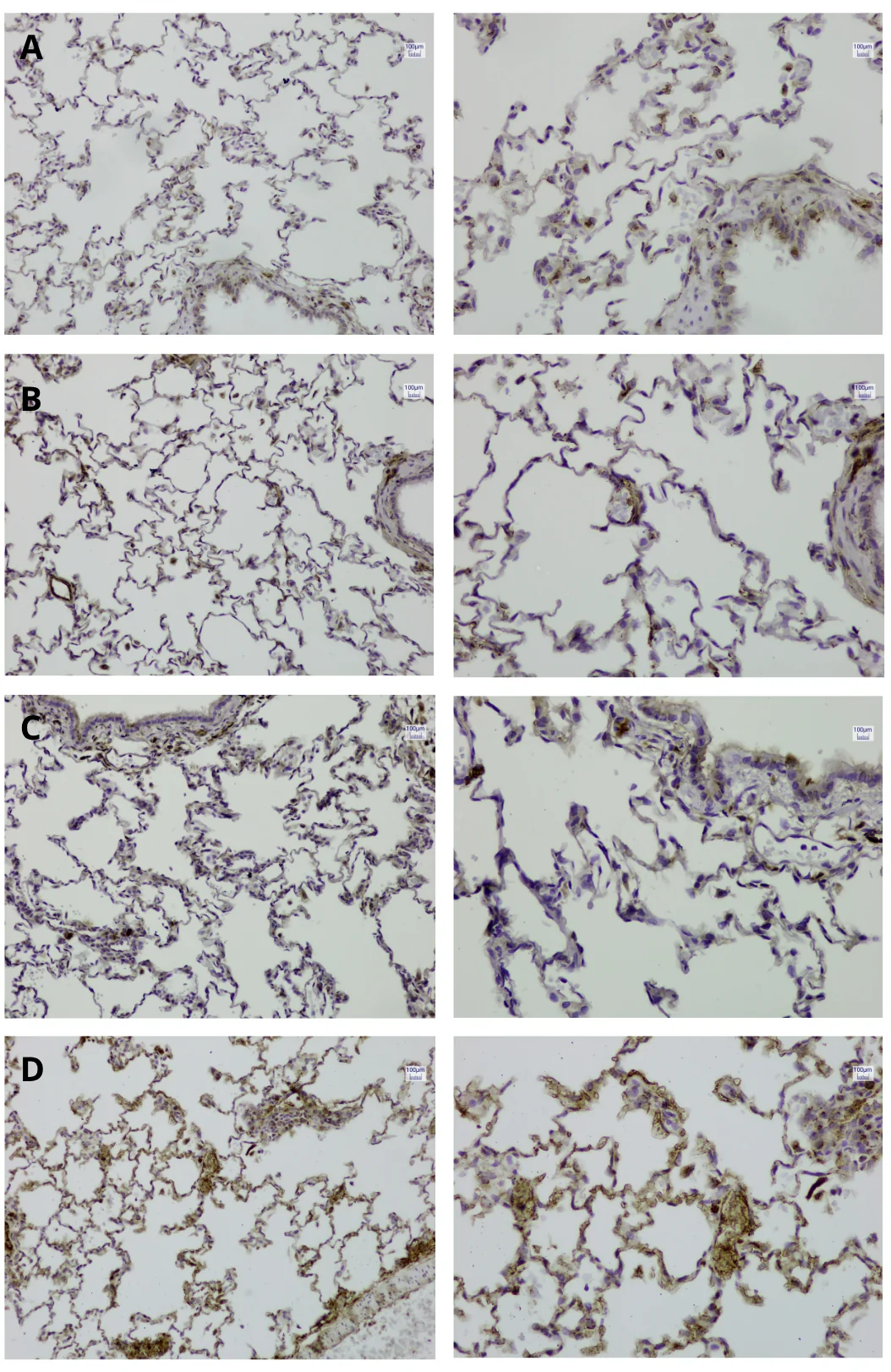
Photomicrographs showing MrgD receptor localization. Positive immunohistochemical reactivity is indicated by brown staining, observed under light microscopy at 20× (**left**) and 40× (**right**) magnifications. (**A**) Control group; (**B**) ALA-P group (asthmatic with prophylactic treatment); (**C**) ALA-T group (asthmatic with therapeutic treatment); (**D**) OVA group (asthmatic without treatment). MrgD receptor immunolabeling was performed in the lung parenchyma using a polyclonal anti-MrgD antibody (dilution 1:200).

**Figure 7:**
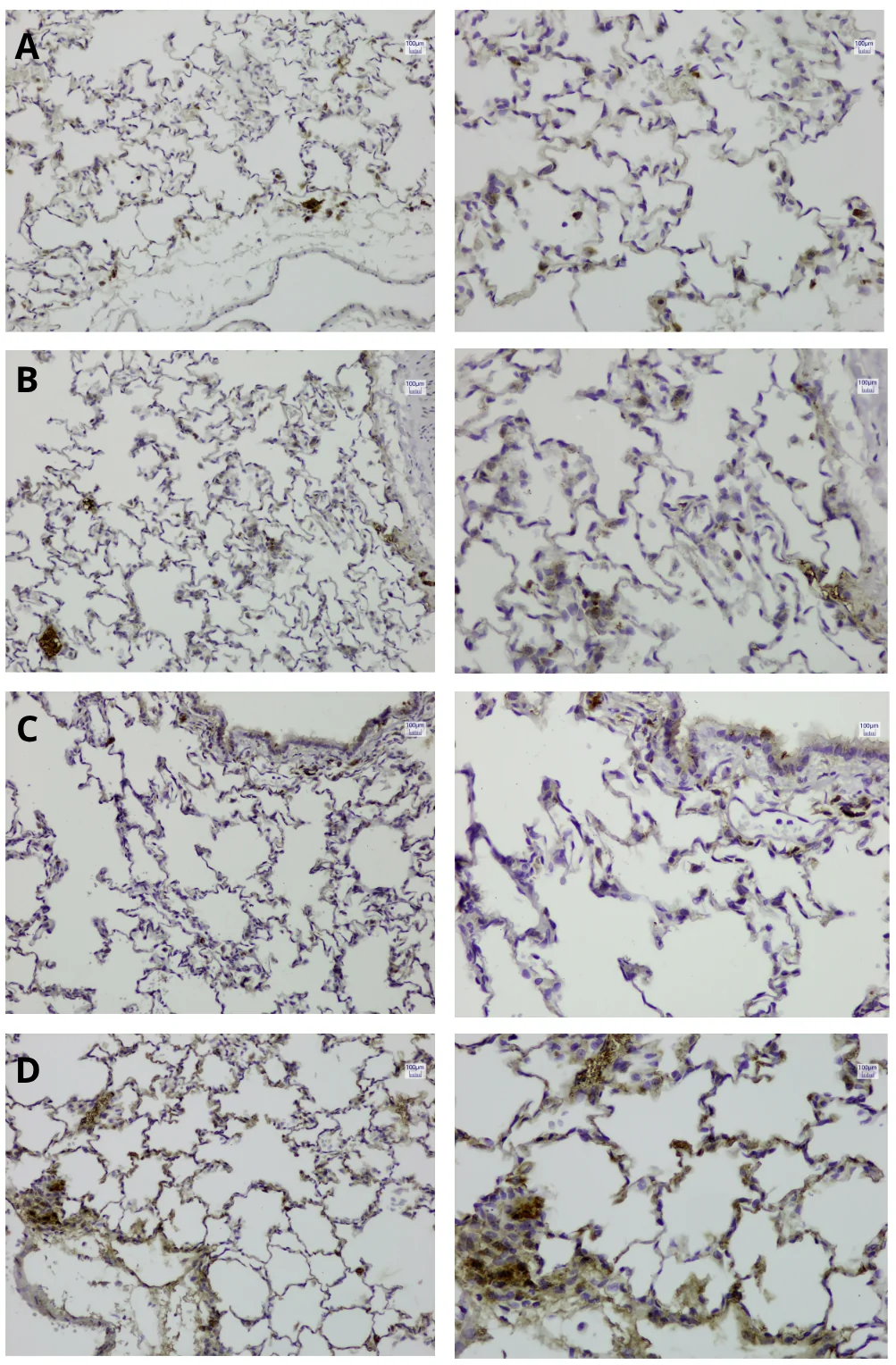
Photomicrographs showing Mas receptor localization. The positive immunohistochemical reactivity is indicated by brown staining, observed under light microscopy at 20× (**left**) and 40× (**right**) magnifications. (**A**) Control group; (**B**) ALA-P group (asthmatic with prophylactic treatment); (**C**) ALA-T group (asthmatic with therapeutic treatment); (**D**) OVA group (asthmatic without treatment). Mas receptor immunolabeling was detected in lung parenchyma using a polyclonal anti-Mas antibody (dilution 1:200).

MrgD and MAS receptor expression in lung tissue were significantly higher in the OVA group compared to all other groups (*p* < 0.0001; Figure 4 and Figure 5), reflecting upregulation of the protective RAS axis during allergic inflammation. Representative photomicrographs illustrating receptor immunostaining are shown in Figure 6 and Figure 7. Both ALA-P and ALA-T groups exhibited significantly elevated MrgD expression relative to CO (*p* < 0.05), with no significant difference between the two treatment groups. This suggests that ALA administration, regardless of timing, amplifies MrgD receptor availability beyond baseline levels. Similarly, both ALA-P (*p* < 0.05) and ALA-T (*p* < 0.01) groups showed significantly higher MAS expression than CO, again with no significant difference between treatment groups. These findings indicate that ALA treatment does not restore receptor expression to control levels but instead sustains and reinforces activation of the protective ACE2/MrgD and ACE2/MAS axes. This sustained activation may underlie the attenuation of airway inflammation and hyperresponsiveness observed in treated animals.

### 3.4. Western Blot

**Figure 8:**
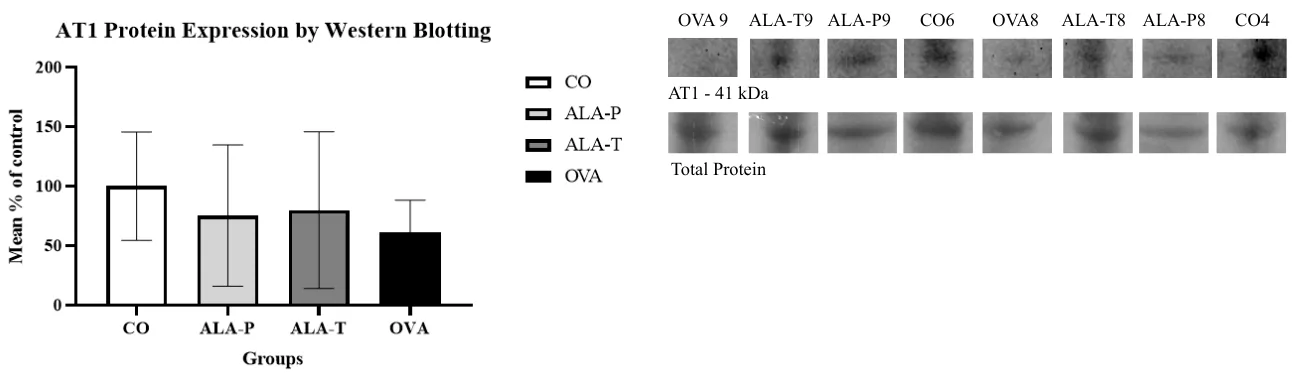
AT1 receptor protein expression measured by Western blotting. The bar graph represents the mean ± SD of AT1 protein levels normalized to total protein content, expressed as percentage relative to the control group (CO). Representative blot images are shown on the right (AT1—41 kDa). CO: control group; ALA-P: prophylactic alamandine-treated group; ALA-T: therapeutic alamandine-treated group; OVA: ovalbumin-induced asthma group. Bars represent the mean ± SD. Differences between groups were analyzed by one-way ANOVA followed by Tukey’s post hoc test.

Regarding AT1 receptor expression, no statistically significant differences were observed among the experimental groups (*p* = 0.6972; Figure 8). Quantitative densitometric analysis of Western blot bands demonstrated uniform levels of AT1 protein expression across all evaluated groups. These findings indicate that neither the asthma induction model nor the ALA treatment significantly altered AT1 receptor expression under the experimental conditions tested.

## 4. Discussion

In this study, ALA treatment appeared to exert potent antiallergic and bronchoprotective effects in an OVA-induced rat model of asthma. These improvements were most pronounced in the therapeutic protocol (ALA-T), suggesting that ALA may retain considerable therapeutic efficacy even when administered after the inflammatory phenotype is established.

The OVA protocol successfully induced airway dysfunction, as evidenced by elevated baseline resistance and the response to methacholine challenge. This profile is consistent with earlier studies demonstrating the reliability of methacholine in eliciting bronchoconstriction and revealing bronchial hyperresponsiveness [7,8,49,50,51]. Therapeutic ALA administration significantly attenuated both basal resistance and methacholine-induced reactivity, restoring values close to those of control animals. These improvements may be attributable to the resolution of chronic airway inflammation, a fundamental driver of asthma pathophysiology [11,52].

The baseline differences observed between our results and previous reports [50,51] may stem from methodological variations, including sensitization intensity, antigen exposure schedules, and strain-specific susceptibility, factors known to influence the severity of airway inflammation [53,54]. In this context, the markedly elevated baseline resistance in the OVA group suggests that our model produced a robust inflammatory state.

Eosinophilic infiltration, a hallmark of allergic asthma, was markedly increased in OVA animals, confirming the successful induction of a Th2-high phenotype [55]. Our results demonstrated that ALA T nearly normalized eosinophil levels, whereas ALA P achieved only a partial reduction. The protective RAS axis may support the resolution of inflammatory responses, including eosinophilic and neutrophilic inflammation [12,33,56,57]. The superior efficacy observed with therapeutic ALA administration suggests that its anti-inflammatory actions may become more pronounced under conditions of active inflammation.

In the present study, Mas and MrgD expression levels were elevated in OVA animals. This likely reflects a compensatory activation of the protective RAS axis in response to chronic inflammation, as previously documented in experimental and human lung disease [24]. Both treatment groups showed reduced receptor expression compared to OVA. However, ALA-T achieved greater normalization, with expression levels closer to those of the CO group, while ALA-P only partially attenuated this upregulation, with expression remaining significantly above control levels.

These findings may suggest that therapeutic ALA administration more effectively addresses the physiological demand for protective RAS signaling. This may reduce the need for endogenous compensatory upregulation to a greater extent than prophylactic treatment. The anatomical distribution of these receptors, Mas in bronchial structures and MrgD in parenchyma [24], may contribute to the distinct expression patterns across groups. Moreover, the potential functional cooperation or heterodimerization between Mas and MrgD [28,29] offers a plausible mechanistic basis for the coordinated receptor modulation observed after treatment.

Although the classical Ang II–AT1 axis is closely implicated in airway hyperresponsiveness and inflammatory remodeling [58,59], no significant differences in AT1 expression were detected among groups. This does not invalidate the functional relevance of AT1, as hyperactivation can occur independently of changes in total protein abundance via increased ligand availability, intracellular signaling alterations, or region-specific modulation not captured in whole-lung homogenates [53,54,58,59].

Our findings indicate that ALA’s protective actions appear to arise primarily from the direct activation of the alternative RAS axis rather than through the suppression of the classical Ang II-AT1 pathway. This selective modulation supports the role of the ALA–MrgD/Mas pathways as a primary counter-regulatory mechanism that restores pulmonary homeostasis without requiring a global downregulation of the classical RAS components.

A similar pattern has been observed in other inflammatory and fibrotic conditions, where Ang II–AT1-driven injury is functionally opposed by ACE2/Ang-(1-7)1 7)/Mas and ALA/MrgD activation, often without global downregulation of AT1 itself [32,60]. For example, Ang-(1-7) or ALA can attenuate lung injury, macrophage-driven inflammation, and Ang II-induced hypertension, primarily through Mas/MrgD-dependent signaling, despite ongoing activity of the classical Ang II–AT1 axis [14,60].

Collectively, the functional, cellular, and molecular data suggest that ALA acts through the coordinated modulation of Mas and MrgD receptors, reducing eosinophilic infiltration and improving airway mechanics. The superior efficacy of therapeutic ALA supports the possibility that its pharmacological actions may be amplified when the inflammatory environment is active, thereby enhancing the engagement of the alternative RAS axis.

Beyond the magnitude of RAS axis engagement, the superior efficacy of the therapeutic regimen carries distinct translational implications. Prophylactic administration models a preventive strategy. This approach may be useful in individuals with known allergen exposure or predictable disease triggers. However, it has limited applicability to the clinical reality of asthma, in which patients typically present after inflammation and airway dysfunction are already established.

In this sense, the therapeutic protocol more closely resembles real-world clinical management. Its ability to reverse airway hyperresponsiveness and eosinophilic infiltration, rather than merely attenuating their progression, suggests a broader role for ALA. The peptide may act not only as an anti-inflammatory agent but also as a disease-modifying intervention capable of resolving an established inflammatory phenotype. This distinction is clinically relevant. Prophylactic strategies are rarely feasible in human asthma management, whereas an agent effective after disease onset would address a more pressing therapeutic need.

While the intracellular signaling pathways downstream of MrgD in the lung remain to be fully elucidated, this functional improvement may involve the activation of AMPK, a key metabolic sensor downstream of the MrgD pathway [38], given that AMPK signaling is a recognized regulator of airway smooth muscle reactivity [61] and inflammatory resolution. Therefore, this pathway emerges as a plausible candidate to link the observed reduction in eosinophilia and the restoration of normal ventilatory mechanics.

These observations align with extensive evidence demonstrating the anti-inflammatory, antifibrotic, antioxidant, and homeostatic roles of the alternative RAS arm across multiple tissues [30,31,32,33,34,35,36,37,38,56,57,62,63]. While our findings provide evidence supporting the efficacy of both ALA treatment regimens, certain methodological considerations must be acknowledged to contextualize the interpretation of these results.

First, the OVA-induced model, despite being well-established and widely used, reproduces an acute allergic response. It does not fully capture the chronic, remodeling-associated features of human asthma. Additionally, its severity varies according to strain and sensitization protocol [53,54], which may influence the magnitude of airway hyperresponsiveness observed across different studies. Therefore, the translatability of these findings to chronic clinical disease should therefore be interpreted with caution.

Second, the assessment of AT1 expression in whole-lung homogenates may mask subtle cell-type-specific changes, particularly in airway epithelium or smooth muscle, where localized RAS modulation might occur.

Third, MrgD and Mas expression were evaluated by immunohistochemistry, which quantifies protein abundance but does not assess receptor functional activity. This limitation includes aspects such as downstream signaling activation, ligand binding, or pharmacological blockade. Consequently, a direct causal link between changes in receptor expression and the functional improvements in airway mechanics cannot be definitively established.

Finally, ALA was administered at a single fixed dose (50 µg/kg/day) and timing based on prior literature rather than a comprehensive dose–response analysis. This study also included only male animals. Therefore, whether lower or higher doses could yield greater or more sustained efficacy, and whether the findings extend to female animals, remains to be determined.

Furthermore, although airway resistance and eosinophilic infiltration jointly confirm successful induction of the allergic phenotype, we did not include molecular confirmation via serum anti-OVA IgE or BALF IL-5. We also did not perform PAS staining to assess goblet cell hyperplasia and mucus plugging, which could provide a direct structural correlate of the increased airway resistance observed. Despite these limitations, the consistency between functional, cellular, and receptor-level outcomes strengthens the overall interpretation. This consistency suggests that ALA meaningfully modulates the inflammatory environment and RAS signaling in allergic asthma.

In summary, both ALA-T and ALA-P treatment attenuated airway hyperresponsiveness, reduced eosinophilic infiltration, and modulated Mas and MrgD receptor expression in this experimental model of allergic asthma. These benefits were most pronounced in the therapeutic regimen, which effectively reverse established airway dysfunction and restore receptor expression toward control levels, whereas ALA-P provided partial but significant attenuation. The absence of changes in AT1 expression suggests that ALA acts primarily through activation of the protective RAS axis rather than via modulation of the classical Ang II–AT1 pathway.

## 5. Conclusions

Our findings indicate that ALA exerts anti-inflammatory and bronchoprotective effects in this OVA-induced asthma model. The therapeutic regimen proved superior to the prophylactic approach by more effectively reversing airway hyperresponsiveness, eosinophilic infiltration, and receptor expression toward control levels. These changes were accompanied by modulation of the MrgD/Mas pathway, without detectable alterations in AT1 expression.

Collectively, these results suggest that the ALA–MrgD/Mas axis may represent a promising target for allergic airway disease, particularly when conventional therapies provide incomplete control. However, further mechanistic and translational research is warranted.

## Data Availability

The original contributions presented in this study are included in the article. Further inquiries can be directed to the corresponding author.

## Abbreviations

The following abbreviations are used in this manuscript:

AHR	Airway hyperresponsiveness
ALA	Alamandine
ALA-P	Prophylactic Alamandine treatment group
ALA-T	Therapeutic Alamandine treatment group
AMPK	5′ AMP-activated protein kinase
ANOVA	Analysis of variance
AT1	Angiotensin II type 1 receptor
BSA	Bovine serum albumin
CO	Control group
Cst	Static compliance
DAB	3,3′-Diaminobenzidine
Est	Elastance
H&E	Hematoxylin and eosin
HPA	Hypothalamic–pituitary–adrenal axis
ICS	Inhaled corticosteroids
MAPK	Mitogen-activated protein kinase
NO	Nitric oxide
OVA	Ovalbumin
PBS	Phosphate-buffered saline
PEEP	Positive end-expiratory pressure
RAS	Renin–angiotensin system
Rn	Airway resistance
SD	Standard deviation
Th2	T-helper lymphocytes type 2
